# m-Health for Burn Injury Consultations in a Low-Resource Setting: An Acceptability Study Among Health Care Providers

**DOI:** 10.1089/tmj.2019.0048

**Published:** 2020-04-16

**Authors:** Anders Klingberg, Hendry Robert Sawe, Ulf Hammar, Lee Alan Wallis, Marie Hasselberg

**Affiliations:** ^1^ Department of Public Health Sciences, Karolinska Institutet, Widerströmska Huset, Stockholm, Sweden.; ^2^ Department of Emeregency Medicine, Muhimbili University of Health and Allied Sciences, Dar Es Salaam, Tanzania.; ^3^ Department of Medical Sciences, Uppsala University, Epihubben, MCT-Huset, Uppsala, Sweden.; ^4^ Division of Emergency Medicine, Faculty of Medicine and Health Sciences, Stellenbosch University, Bellville, South Africa.; ^5^ Division of Emergency Medicine, University of Cape Town, Cape Town, South Africa.

**Keywords:** emergency medicine/teletrauma, m-health, technology, telemedicine

## Abstract

***Introduction:*** The rapid adoption of smartphones, especially in low- and middle-income countries, has opened up novel ways to deliver health care, including diagnosis and management of burns. This study was conducted to measure acceptability and to identify factors that influence health care provider's attitudes toward m-health technology for emergency care of burn patients.

***Methods:*** An extended version of the technology acceptance model (TAM) was used to assess the acceptability toward using m-health for burns. A questionnaire was distributed to health professionals at four hospitals in Dar Es Salaam, Tanzania. The questionnaire was based on several validated instruments and has previously been adopted for the sub-Saharan context. It measured constructs, including acceptability, usefulness, ease of use, social influences, and voluntariness. Univariate analysis was used to test our proposed hypotheses, and structural equation modeling was used to test the extended version of TAM.

***Results:*** In our proposed test-model based on TAM, we found a significant relationship between compatibility—usefulness and usefulness—attitudes. The univariate analysis further revealed some differences between subgroups. Almost all health professionals in our sample already use smartphones for work purposes and were positive about using smartphones for burn consultations. Despite participants perceiving the application to be easy to use, they suggested that training and ongoing support should be available. Barriers mentioned include access to wireless internet and access to hospital-provided smartphones.

## Introduction

The cost of smartphones relative to their usefulness and ease of use has spawned an interest in these devices in health care, especially in resource-poor countries.^1^ Being equipped with large screens and cameras makes smartphones particularly useful in specialties with a prominent visual component,^2,3^ such as acute burn care.^4–6^ In fact, smartphones are already used by health professionals for burn care, not only to discuss patient findings via text and voice calls but also by image or video exchange.^5–7^ Studies have demonstrated that burns specialists can effectively diagnose burns by viewing images or video of the burn sent by the frontline provider.^8,9^ Two studies from South Africa describe their experience using WhatsApp to discuss referrals between health centers and specialist burns units: in Cape Town, the use of WhatsApp reduced unnecessary referrals and outpatient visits,^5^ while in Durban, not only did it reduce unnecessary referrals but it also changed management in two-thirds of patients.^6^ However, there are some drawbacks to using commercial chat services, not least of which are the privacy and informed consent.^10,11^ The information sent via these apps may also be hard to incorporate into existing electronic medical record systems.^11^ An alternative would be to develop communication apps that address these issues. However, before implementing a new technology involving a new way of working, user acceptance should be evaluated.

**Fig. 1.**
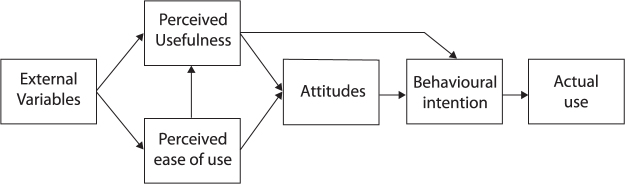
Technology acceptance model by Davis (1989).

Technology acceptance is referred to as an individual's intentional or voluntary use of a technology.^12^ Adoption of new technologies is influenced by an individual's attitudes toward the technology.^12^ Attitude refers to the degree to which a person forms positive or negative perceptions about a particular behavior.^13^ This correlation between attitude and intention has been extensively validated in studies on the adoption of various technological products.^14,15^ Several theories have been proposed to explain why individuals choose to use new technologies. The first theory developed to explain users' intention to use (or to reject) new technologies was the technology acceptance model (TAM).^12^ TAM (*Fig. 1*) was adapted from the theory of reasoned action, which explains behavior as a result of beliefs toward this behavior.^12^ TAM posits that usage is predicted by a user's behavioral intention (BI), which is influenced by attitudes toward this behavior. Users form their attitudes depending on the perceived usefulness and perceived ease of use of the new technology. Perceived usefulness also has a direct influence on BI.^12^ Furthermore, the influence of perceived ease of use on attitudes has been shown to be weak and instead influences attitudes through perceived usefulness.^16^ Other factors affecting attitudes and subsequently intention to use are, for example, compatibility, image, voluntariness,^17^ self-efficacy, anxiety,^18^ and facilitating conditions.^19^

In this study, we focus on the attitudes toward using m-health technology for the diagnosis and management of patients with burns in a resource-constrained setting. According to the latest Global Health Estimates study, in 2016, burns were responsible for 153,000 deaths,^20^ and it is estimated that 10 million disability-adjusted life years are lost due to burns each year.^21^ In addition, the vast majority of these burns (94%) occur in low- and middle-income countries (LMIC)^20^ where specialist burn care is often limited or nonexistent.^22,23^ Providing timely and appropriate burn care is essential to minimize complications, and reduce morbidity and mortality.^24^ While there has been a downward trend in burn mortality worldwide since the turn of the century, death as a result of burns has increased in the African WHO region.^20^ According to Global Health Estimates, Tanzania has one of the highest rates of burn injury death in the world, especially among young children.^20^ In addition, Tanzania lacks dedicated burns units, and therefore, severe cases are often managed in the surgical ward or ICU, which are also limited in infrastructure, personnel, and resources.^25^ There is also a lack of trained staff in burn care as well as lack of medical equipment and medications to treat burns.^26^ While m-health technology cannot solve these problems alone, it can support the coordination and utilization of the existing human, material, and financial resources. Consequently, the aim was to identify factors that influence health care provider's attitudes toward m-health technology for emergency burn care. A secondary aim was to assess if these factors differ depending on age, sex, type of facility, burn care experience, and occupation.

## Materials and Methods

The study was a cross-sectional questionnaire-based survey of health providers working in the emergency department and who are involved in the management of burn patients.

### The Vula application

**Fig. 2.**
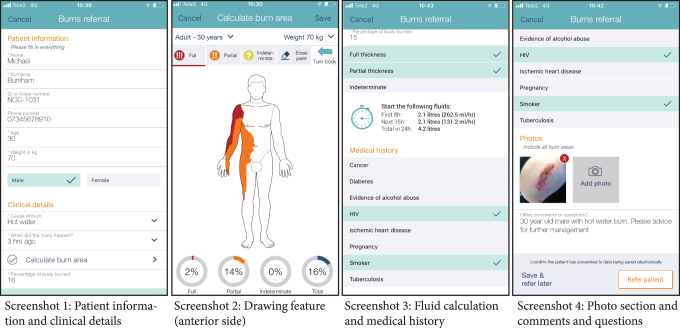
Screenshots from the Vula app. Color images are available online.

The smartphone application (app) of interest for this study is the Vula medical referral app for burn referrals. The purpose of the app is for clinicians to quickly document patient and clinical information, send the information to a burns specialist who will reply with management and referral advice. The interface contains a structured form for the user to fill out information such as patient and injury data (*Fig. 2*, screenshot 1), and a drawing feature where the user can depict burn surface and depth (*Fig. 2*, screenshot 2). This will also calculate the surface of the burn as well as required fluids based on the Parkland formula (*Fig. 2*, screenshot 3). An important feature is also the possibility to take and send photos of the burn injury and to add additional comments and questions (*Fig. 2*, screenshot 4). When a referral has been sent, a chat function within the app allows the referring clinician and the consultant to further communicate. The usability of the app has been previously evaluated with South African users, both emergency doctors and burns specialists.^27^ In another study by Blom et al., South African burns and emergency care specialists believed the app would streamline the diagnostic process, improve both triage and referrals, and be a more secure option for remote diagnosis compared with current practices.^28^

### Setting and participants

The study was conducted at Muhimbili National Hospital, and in three regional referral hospitals: Temeke, Amana, and Mwananyamala Regional Referral Hospitals in Dar Es Salaam, Tanzania. The target population of the study was health providers working in the emergency departments at the four facilities. These included doctors, nurses, health attendants, assistant medical officers, clinical officers, and medical students.

### Data collection

A questionnaire was distributed to the participants during working hours to fill out and participation was voluntary. Participants were given a short introduction to the purpose of the study and the functionality of the app. The questionnaire was adapted from Kifle et al.,^29^ and included 49 questions measuring 11 different constructs. Their definitions and the full set of questions are presented in *Table 1*. The wording of the original items was adapted to reflect the technology, task, and context of this study. Variables were measured on a 7-point Likert scale ranging from 1 = “strongly disagree” to 7 = “strongly agree.”

**Table 1.** Constructs, Definitions, and Measurement Items

**CONSTRUCT**	**MEASUREMENT ITEM**	
Perceived ease of use (Davis)^12^ TAM [The degree to which a person believes that using a particular system would be free of effort]	(1)	Learning to operate a smartphone would be easy for me
	(2)	Learning to operate an app like this would be easy for me
	(3)	It would be easy for me to become skillful at using such an app
	(4)	My interaction with an app like this would be clear and understandable
Perceived usefulness (Davis)^12^ TAM [The degree to which a person believes that using a particular system would enhance his or her job performance]	(5)	Using an app like this could improve the care I give to my patients
	(6)	If I were to use the app I could see more patients in the emergency room
	(7)	Using an app like this would increase my efficiency
	(8)	This app would be an improvement in the area where I see most of my patients (e.g., emergency room)
	(9)	I would find an app of that kind useful in my job
	(10)	Using such an app would enable me to accomplish some tasks more quickly
Compatibility (Moore and Benbasat)^17^ IDT [The degree to which an innovation is perceived as being consistent with the existing values, needs, and past experiences of potential adopters]	(11)	Using an app like this would be compatible with most aspects of my work
	(12)	I think that using an app like this would fit well with the way I like to work
Image (Moore and Benbasat)^17^ IDT [The degree to which use of an innovation is perceived to enhance one's image or status in one's social system]	(13)	If I were to use such an app I would gain more prestige among my peers
	(14)	Using such an app would be a status symbol in my department
	(15)	People in my organization who would use an app of that kind would have more prestige than those who do not
Self-Efficacy (Compeau and Higgins)^18^ IDT [Judgment of one's ability to use a technology to accomplish a particular job or task]		I could use the app…
	(16)	…If I had used similar apps before
	(17)	…Even if I had never used an app like it before
	(18)	…If I only had the built-in “help” function for assistance
	(19)	…Even if there was no one around to tell me what to do as I go
	(20)	…If I had seen someone else using it before
	(21)	…If someone showed me how to use the app beforehand
	(22)	…if I had a lot of time to use the application
Voluntariness (Moore and Benbasat)^17^ IDT [The degree to which use of the innovation is perceived as being voluntary, or of free will]	(23)	The department head does not require me to use apps like this
	(24)	Although it might be helpful, using an app like this is certainly not compulsory in my job
Behavioural intention to adopt (Fishbein and Ajzen)^30^ TPB [An individual's positive or negative feelings (evaluative effect) about performing the target behavior]	(25)	I intend to use an app like this when it becomes available
	(26)	Over the ensuring months (if possible) I plan on experimenting with the app
	(27)	Over the ensuring months (if possible) I plan to regularly use such an app
Anxiety (Compeau and Higgins)^18^ IDT [Evoking anxious or emotional reactions when it comes to performing a behavior]	(28)	I am concerned about possible liability issues associated with the use of this app
	(29)	I do not like the loss of personal contact associated with using apps like this
	(30)	More research is needed on the effectiveness of apps like this before I would refer patients using the app
	(31)	If additional credentialing and licensure procedures were required that would discourage me from using apps like this
	(32)	I think an expert can adequately make an assessment of the patient when not being physically present
	(33)	I feel apprehensive about using an app like this
	(34)	It scares me to think that I could lose a lot of information using such an app by hitting the wrong button
	(35)	I hesitate to use such an app for fear of making mistakes I cannot correct
Social influences (Fishbein and Ajzen)^30^ TPB [The person's perception that most people who are important to him think he should or should not perform the behavior in question]	(36)	People who influence my behavior may think that I should use an app like this
	(37)	People who are important to me at work may think that I should use an app like this
	(38)	The senior management of this facility will be helpful in the use of such an app
	(39)	In general, the facility management will be supportive of the use of an app of this kind
	(40)	In general, the district health services management will be supportive of the use of such an app
Facilitating conditions (Thompson et al.)^19^ TPB [Objective factors in the environment that observers agree make an act easy to accomplish. For example, provision of support for users of personal computers]	(41)	I have the resources necessary to use such an app
	(42)	I have the knowledge necessary to use an app like this
	(43)	An app like this is not compatible with the way we work
	(44)	A specific person (or group) should be available for assistance with difficulties concerning an app like this
Attitude toward a behavior (Fishbein and Ajzen)^30^ TPB [An individual's positive or negative feelings (evaluative effect) about performing the target behavior]	(45)	Using an app like this for burn emergency care is a good idea
	(46)	Using an app like this where I work is a good idea
	(47)	An app like this would make work more interesting
	(48)	Working with such an app would be fun
	(49)	I would like working with such an app

TAM, technology acceptance model; IDT, innovation diffusion theory; TPB, theory of planned behaviour.

### Data analysis

To explain the intention to use a smartphone app for burn care, different constructs based on theories from technology acceptance and behavioral change were included. *Table 2* presents the proposed hypotheses. We used structural equation modeling (SEM) to test the hypotheses presented in *Table 2* and to examine the model proposed by Kifle et al.^29^ SEM is a multivariate statistical analysis technique that is used for analyzing structural relationships between measured variables and latent constructs. We also performed multigroup SEM analyses for the univariate hypotheses, with groups being men/women, referral/referring, age group (24–29/30+), doctor/nurse, and self-rated burn care experience. In these analyses, the item loadings were constrained to be similar for the two categories being tested. All analyses were performed using Stata 13.

**Table 2.** Hypotheses Included in the Analysis

**HYPOTHESIS**	**DEFINITION**
H1	Computer self-efficacy is positively related to their perception of ease of use of the app
H2	Facilitating conditions are positively related to their attitude toward the app
H3	Perceived compatibility is positively related to their perception of the usefulness of the app
H4	Perceived ease of use of the app is positively related to their perception of its usefulness
H5	Image is positively related to their attitude toward the app
H6	Voluntary use of the app is positively related to their attitude toward the app
H7	Social influences are positively related to their attitude toward the app
H8	Anxiety toward the use of the app is negatively related to their attitude toward the app
H9	Perceived ease of use of the app is positively related to their attitude toward the app
H10	Usefulness of the app is positively related to their attitude toward the app

### Ethical considerations

The study was approved by the National Institute for Medical Research, and the Office of the Director of Research and Publications at Muhimbili University of Health and Allied Sciences. Permission was also sought from the administration of each of the participating hospitals. Participation was voluntary, and written consent for participation was obtained for each health worker.

## Results

### Attitudes toward the smartphone app

Fifty-nine respondents were included in the analysis. All except three owned smartphones. Smartphones were used for several purposes, including looking up information on diagnosis and drug dosage, medical reference and calculation apps, communication and data collection. Most indicated that they had either moderate or extensive experience in burn care. The participants rated high on concepts such as usefulness, attitude, and BI, suggesting that they were positive toward using the app (*Table 3*). Cronbach's alpha was used to test reliability and was found to be satisfactory for all (above 0.70) constructs except compatibility, self-efficacy, voluntariness, anxiety, and facilitating conditions. Characteristics of the participants are presented in *Table 4*.

**Table 3.** Number of Items, Item Mean, and Reliability Statistics (Cronbach's Alpha) of Each Construct

**CONSTRUCT**	**NO. OF ITEMS**	**ITEM MEAN**	**CRONBACH'S ALPHA**
Perceived ease of use	4	6.44	0.89
Perceived usefulness	6	6.16	0.89
Compatibility	2	6.03	0.53
Image	3	5.44	0.93
Self-efficacy	7	3.84	0.58
Voluntariness	2	3.49	0.60
BI to adopt	3	5.87	0.87
Anxiety	8	4.40	0.48
Social influences	5	5.18	0.80
Facilitating conditions	4	4.41	0.10
Attitude toward using technology	5	6.38	0.86

**Table 4.** Descriptive Statistics of Study Sample (*n* = 59)

**CHARACTERISTICS**	**N (59) (%)**
Gender	
Male	21 (35.6)
Female	38 (64.4)
Facility	
Referral	30 (50.8)
Referring	29 (49.2)
Occupation	
Physician	28 (49.1)
Nurse	21 (36.8)
Other health profession^a^	8 (14.0)
Smartphone use	
Yes	56 (94.9)
No	3 (5.1)
Experience in emergency care	
<1 year	5 (10.2)
2–3 years	17 (34.7)
>3 years	27 (55.1)
Burn experience	
None	0 (0.0)
Minimal	3 (5.8)
Moderate	34 (65.4)
Extensive	15 (28.8)

^a^ Medical students, health attendants, clinical officers, and assistant medical officers.

### Test of hypotheses

*Table 5* shows the proposed hypotheses by gender, type of facility, age, profession, and experience in burn care. We found correlations between 8 of the 11 proposed hypotheses: CO-PU, PEOU-PU, IM-ATT, SI-ATT, ANX-ATT, PEOU-ATT, PU-ATT, and ATT-BI. When we tested if there were any differences between gender, type of facility, between doctors and nurses, and their experience in burn care, we found differences between men and women in PU-ATT, and between referral and referring hospitals in the constructs CO-PU and IM-ATT. Because of the small sample size, many constructs did not converge in the analysis.

**Table 5.** Univariate Analysis of Relationships Between Constructs and Differences Between Gender, Type of Hospital, Age, Occupation, and Self-Rated Experience in Burn Care

**PATH**	**STANDARDIZED**	**ALL (59)**	***p***	**MEN (21)**	**WOMEN (38)**	***p***	**REFERRAL (30)**	**REFERRING (29)**	***p***	**24–29**	**30+**	***p***	**DOCTOR (30)**	**NURSE AND OTHERS (27)**	***p***	**EXPERIENCED**	**INEXPERIENCED**	***p***
SE -> PEOU	Yes	−0.14	0.36	−0.16	−0.06	1.00	0.07	−0.29	0.99	−0.20	−0.12	1.00	0.08	−0.51	1.00	NA	NA	NA
	No	−0.08	1.00	−0.06	−0.03	1.00	0.01	−0.30	0.99	−0.09	−0.09	1.00	0.03	−0.44	1.00	NA	NA	NA
FC -> PEOU	Yes	0.42	0.33	NA	NA	NA	NA	NA	NA	NA	NA	NA	NA	NA	NA	NA	NA	NA
	No	0.22	0.99	NA	NA	NA	NA	NA	NA	NA	NA	NA	NA	NA	NA	NA	NA	NA
CO -> PU	Yes	0.94	**0**	NA	NA	NA	0.89	0.96	**0.03**	NA	NA	NA	NA	NA	NA	NA	NA	NA
	No	0.95	**0.01**	NA	NA	NA	0.43	1.21	**0.03**	NA	NA	NA	NA	NA	NA	NA	NA	NA
PEOU -> PU	Yes	0.70	**0.00**	0.47	0.78	1.00	0.92	0.60	0.99	0.62	0.75	1.00	0.58	0.85	1.00	0.91	0.43	0.99
	No	0.38	1.00	0.24	0.43	1.00	0.43	0.39	0.99	0.41	0.46	1.00	0.16	0.24	1.00	0.42	0.24	0.99
IM -> ATT	Yes	0.55	**0.00**	0.48	0.57	0.12	0.41	0.83	**0.00**	0.53	0.58	0.62	0.53	0.62	0.19	0.60	0.47	0.51
	No	0.41	**0.00**	0.22	0.48	0.12	0.16	0.89	**0.00**	0.37	0.45	0.62	0.29	0.53	0.19	0.45	0.34	0.51
VO -> ATT	Yes	−0.26	0.13	−0.34	−0.24	1.00	−0.39	−0.23	1.00	−0.34	−0.17	1.00	−0.40	−0.18	1.00	−0.29	−0.20	1.00
	No	−0.16	1.00	0.11	−0.15	1.00	−0.11	−0.17	1.00	−0.17	−0.09	1.00	−0.16	−0.11	1.00	−0.14	−0.09	1.00
SI -> ATT	Yes	0.65	**0.01**	0.39	0.63	0.07	0.56	0.52	0.17	0.61	0.51	0.77	0.36	0.79	0.07	0.48	0.64	0.58
	No	0.65	**0.01**	0.27	0.85	0.07	0.36	0.77	0.17	0.70	0.60	0.77	0.31	1.14	0.07	0.55	0.70	0.58
ANX -> ATT	Yes	0.36	**0.02**	0.11	0.42	0.99	0.15	0.45	1.00	0.56	0.30	0.33	0.11	0.40	0.29	0.17	0.75	0.07
	No	0.47	**0.04**	0.09	0.70	0.99	0.09	0.69	1.00	0.80	0.35	0.33	0.11	0.54	0.29	0.22	1.00	0.07
PEOU -> ATT	Yes	0.58	**0.00**	0.80	0.68	1.00	0.84	0.63	1.00	0.58	0.63	1.00	0.64	0.68	NA	0.70	0.45	1.00
	No	0.83	**0.00**	0.63	0.99	1.00	1.05	1.02	1.00	0.79	0.78	1.00	1.05	0.80	NA	1.00	0.58	1.00
PU -> ATT	Yes	0.83	**0.00**	0.81	0.84	**0.03**	0.97	0.72	0.48	0.90	0.81	NA	0.99	0.64	1.00	0.82	0.90	0.99
	No	1.45	**0.00**	0.95	1.62	**0.03**	1.70	1.43	0.48	0.85	0.77	NA	0.75	0.71	1.00	1.00	0.88	0.99
ATT -> BI	Yes	0.41	**0.00**	0.62	0.36	0.73	0.48	0.40	0.97	0.78	0.52	0.65	0.86	0.51	0.74	0.43	0.76	0.50
	No	0.44	**0.00**	0.47	0.37	0.73	0.40	0.41	0.97	0.81	0.67	0.65	0.95	0.82	0.74	0.64	0.87	0.50

Numbers in bold indicate significance at *p* < .05.

SE, self-efficacy; PEOU, perceived ease of use; FC, facilitating conditions; CO, compatibility; PU, perceived usefulness; IM, image; ATT, attitudes; VO, voluntariness; SI, social influence; ANX, anxiety.

### Test of the proposed model

**Fig. 3.**
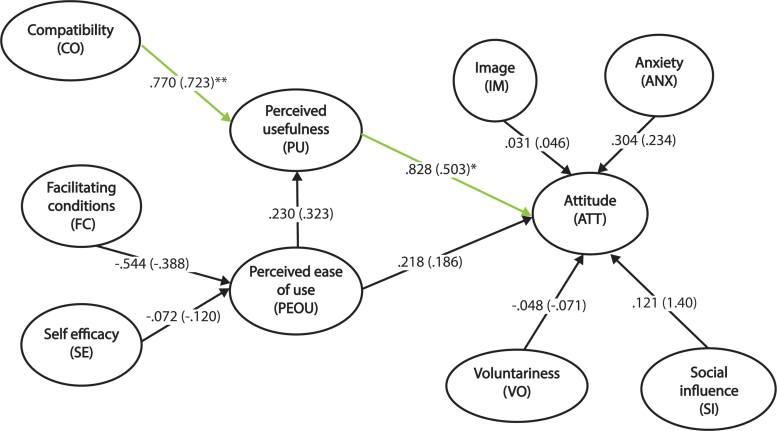
Hypothesis model *significant at 0.05 and **significant at 0.01. Color images are available online.

The results from testing our modified version of the proposed model by Kifle et al. can be found in *Figure 3*. We found significant correlations between Compatibility—Perceived usefulness and Perceived usefulness—Attitude.

### Comments from participants

The most common remarks were related to facilitating conditions such as access to wireless internet within the hospital, training on how to use the app, and that smartphones or tablets should be provided by the hospital. There were also concerns about the privacy issues using the app, especially when taking images of patients. The users were only briefly introduced to the app and its functionality, but still had comments about features or information that they thought should be in the app.

## Discussion

The usefulness of a technology has been found to be one of the strongest predictors of technology acceptance.^31,32^ Our results show that the participants believed that the system would be very useful and that using the app would enhance their ability to provide better care to patients with burns and doing so in a more efficient manner. According to our results, the vast majority of the participants reported moderate or high experience in burn care. Bearing in mind that burn injuries are commonly seen at these hospitals, this finding is unsurprising. There are also difficulties to provide quality care in this setting due to lack of resources such as dressing materials and also lack of personnel, lack of training in burn care, and poor teamwork.^26^ We also found that the correlation between usefulness and attitudes was stronger for women. However, this result has to be interpreted with caution, since there might be other confounding factors that we were unable to adjust for due to the small sample size. Still, research on the role of gender has shown that gender is an influencing factor in technology acceptance, where men are more adept users of technology information systems.^33^

The participants were only briefly introduced to the app but still had comments about features that should be included. This highlights the importance of engaging all indented users before development and implementation to make a product that users find useful.

In addition, there was a significant relationship between compatibility and perceived usefulness of the app, meaning that using an app for burn consultations was seen to be compatible with the way health workers like to work. First, the high number of patients with burn injuries indicates that there is a need for consultation support which the app offers. Second, almost all of the participants owned a smartphone and said they already used it for work purposes, which suggests that they would also use their smartphone for burn consultations.

In line with previous studies, we found a positive correlation between perceived ease-of-use and usefulness.^16,34^ Perceived ease of use of a new technology has been found to influence attitudes and BI, however, this correlation is often weaker than the influence of perceived usefulness on attitude.^32,35^ Many studies have found that ease of use does not directly affect attitudes or BI but is mediated through perceived usefulness.^35^ However, even if a technology is thought to be useful, it also needs to be easy to use to be useful. However, while the app in this study specifically targets burn injuries, with specific functionalities related to burns, there are other ways of performing the same task that may be perceived as easier, such as calling, e-mailing, or sending a text message. A study from Cape Town suggests that WhatsApp is preferred because of its ease of use.^5^ However, Nikolic et al. raise the concern that the widespread use of WhatsApp may hinder the introduction of other apps for communication in health care.^36^ Therefore, ease-of-use becomes highly relevant when developing applications such as the one described in this study.

In addition, facilitating conditions is an important factor, especially in resource-limited settings. Facilitating conditions measure the users' perception regarding the support and necessary infrastructure to use the new technology. We did not find support for the hypothesis that facilitating conditions is related to the perceived as ease of use of the app. While the app was perceived useful and easy to use, factors in the Tanzanian context may hinder its use. For example, access to reliable internet connections was the most frequent comment among users followed by the need for training, which are both facilitating conditions.

The relatively low score on the construct “self-efficacy” indicates that the participants may not feel very comfortable using the app without proper training and familiarization of the app. Furthermore, while most users in our sample reported owning a smartphone, some mentioned that smartphone ownership might still be a barrier. These concerns were also reflected in the question “I have the resources necessary to use such an app” where almost half of the participants rated this item somewhere between neutral and strongly disagree. Although no participants explicitly mentioned the cost of using the app, health workers might be reluctant to use their own devices due to the cost of the data. A study from Malawi and Ghana raises the concern that the cost of “informal m-health” is mainly borne by the health workers themselves.^37^ The implication of this is that different barriers to successful implementation have to be recognized at an early stage.

### Additional factors influencing acceptability

Despite being frequently used, the TAM has been criticized for its limited explanatory and predictive power as well as practical value. This has led to several iterations and extensions of the original model with added constructs that are thought to capture other explanatory factors. We examined several factors and their influence on attitude: image, social influence, anxiety, and voluntariness.

The construct image, that is, the degree to which use of an innovation is perceived to enhance one's image or status in one's social system,^17^ was found to be positively correlated with a positive attitude toward the app, and this correlation was significantly stronger among referring hospitals. These differences could not be explained by gender, profession, age, or other variables. We can only speculate why the participants thought the app would increase their status and image. However, it might not have to do with the use of the app itself but rather being given a tool to carry out the task of managing burns in a proper way. A study from Uganda found that village health workers using a teleconsultation system for maternal and newborn care felt recognized as important stakeholders.^38^

We also found that social influence was positively correlated with their attitude toward the app. For a physician or nurse, social influence to use a new technology might come from the patients and their families, coworkers, managers, or governing bodies. A study from rural Bangladesh found that “social reference,” that is, when people are influenced by important peers, had the strongest impact on attitudes toward e-health.^39^ In our study, there were no differences between referral and referring hospitals in how much social influence affected attitudes. However, the correlation was stronger among women compared with men, and for nurses compared with physicians, but did not reach significance (*p* 0.07 and *p* 0.07, respectively). The notion that women are more affected by social influence has been suggested earlier by Venkatesh and Davis.^40^ This finding also has implications for successful implementation. In a systematic review of adoption of information and communication technology (ICT) in health care, they suggest to identify and support key staff to lead and encourage the use of a new ICT.^31^

While the app is meant to facilitate diagnosis and management of care of patients with burn, there are several other problems in the Tanzanian setting that hinders the delivery of adequate care. For example, a study of the implementation of a burn course in Tanzania found that health workers faced several issues in burn care such as lack of appropriate dressing, lack of training in burn care, problems with detecting infections, as well as maintaining a sterile environment.^26^ This is, unfortunately, the reality in many LMIC where many hospitals are capable of initial burn management and basic resuscitation but lack the capacity to provide advanced burn care.^22^

### Strength and limitations

The strengths of this study include a diverse sample, both in terms of professional background and type of hospital. Some limitations need to be mentioned. First, the study setting was limited to Dar Es Salaam, Tanzania, and therefore, health workers' perceptions may differ from those working in rural communities where the m-health system is likely to be used. Even though we have used adequate methods for taking into consideration a small sample size, some of the results need to be interpreted with caution.

## Conclusion

Most health workers were positive toward using a smartphone app for burn consultations and referrals. Participants thought that the app would be useful and believed it would be easy to use.

Social influence was correlated with positive attitudes, and this correlation was stronger for women. However, the main barriers to utilization included access to internet, smartphones, and technical support. Since this study focused on *acceptability* before using the app, it is important to conduct further studies once the system has been implemented to assess the actual *acceptance* of the app. Further studies should also identify key users such as a head nurse who can encourage use, and also assist with the introduction and ongoing usage.

### Availability of data and material

The data material used to support the results of this study is available from the corresponding author on request.

## References

[1] Betjeman TJ, Soghoian SE, Foran MP mHealth in Sub-Saharan Africa. Int J Telemed Appl 2013:1–7 10.1155/2013/482324 PMC3867872 24369460

[2] Sikka N, Carlin KN, Pines J, Pirri M, Strauss R, Rahimi F The use of mobile phones for acute wound care: Attitudes and opinions of emergency department patients. J Health Commun 2012;17(SUPPL. 1):37–43 22548597 10.1080/10810730.2011.649161

[3] Perera CM, Chakrabarti R A Review of m-Health in Medical Imaging. Telemed J E Health 2015;21:132–137 25584516 10.1089/tmj.2013.0330

[4] Wallis LA, Fleming J, Hasselberg M, Laflamme L, Lundin J A smartphone app and cloud-based consultation system for burn injury emergency care. PLoS One 2016;11:1–19 10.1371/journal.pone.0147253 PMC4769217 26918631

[5] Martinez R, Rogers AD, Numanoglu A, Rode H The value of WhatsApp communication in paediatric burn care. Burns 2018;44:947–955 29395403 10.1016/j.burns.2017.11.005

[6] den Hollander D, Mars M Smart phones make smart referrals: The use of mobile phone technology in burn care–A retrospective case series. Burns 2017;43:190–194 27575675 10.1016/j.burns.2016.07.015

[7] Boccara D, Bekara F, Soussi S Ongoing development and evaluation of a method of telemedicine: Burn care management with a smartphone. J Burn Care Res 2018;39:1–5 29789857 10.1093/jbcr/irx022

[8] Boissin C, Laflamme L, Wallis L, Fleming J, Hasselberg M Photograph-based diagnosis of burns in patients with dark-skin types: The importance of case and assessor characteristics. Burns 2015;41:1253–1260 25716764 10.1016/j.burns.2014.12.014

[9] Cai LZ, Caceres M, Dangol MK, et al. Accuracy of remote burn scar evaluation via live video-conferencing technology. Burns 2016:1–8 10.1016/j.burns.2016.11.006 27931764

[10] Kamel Boulos MN, Giustini DM, Wheeler S Instagram and WhatsApp in health and healthcare: An overview. Future Internet 2016;8:1–14

[11] Mars M, Escott R WhatsApp in clinical practice: A literature review. Stud Health Technol Inform 2016;231:82–90 27782019

[12] Davis FD Perceived usefulness, perceived ease of use, and user acceptance of information technology. MIS Q 1989;13:319–340

[13] Ajzen I, Fishbein M Attitudes and normative beliefs as factors influencing behavioral intentions. J Pers Soc Psychol 1972;21:1–9

[14] Moon BC, Chang H Technology acceptance and adoption of innovative smartphone uses among hospital employees. Healthc Inform Res 2014;20:304–312 25405067 10.4258/hir.2014.20.4.304 PMC4231181

[15] Hu PJ, Chau PYK, Liu Sheng OR, Tam KY Examining the technology acceptance model using physician acceptance of telemedicine technology. J Manag Inf Syst 1999;16:91–112

[16] Holden RJ, Karsh BT The technology acceptance model: Its past and its future in health care. J Biomed Inform 2010;43:159–172 19615467 10.1016/j.jbi.2009.07.002 PMC2814963

[17] Moore GC, Benbasat I Development of an instrument to measure the perceptions of adopting an information technology innovation. Inf Syst Res 1991;2:192–222

[18] Compeau DR, Higgins CA Computer self-efficacy: Development of a measure and initial test. MIS Q 1995;19:189–211

[19] Thompson RL, Higgins CA, Howell JM Personal computing: Toward a conceptual model of utilization. MIS Q 1991;15:125

[20] *Global Health Estimates 2016: Deaths by Cause, Age, Sex, by Country and by Region, 2000–2016.* Geneva, Switzerland: World Health Organization, 2018

[21] *Global Health Estimates 2016: Disease Burden by Cause, Age, Sex, by Country and by Region, 2000–2016*. Geneva, Switzerland: World Health Organization, 2018.

[22] Gupta S, Wong EG, Mahmood U, Charles AG, Nwomeh BC, Kushner AL Burn management capacity in low and middle-income countries: A systematic review of 458 hospitals across 14 countries. Int J Surg 2014;12:1070–1073 25152443 10.1016/j.ijsu.2014.08.353

[23] Charles AG, Gallaher J, Cairns BA Burn care in low- and middle-income countries. Clin Plast Surg 2017;44:479–483 28576236 10.1016/j.cps.2017.02.007

[24] Alharbi Z, Piatkowski A, Dembinski R, et al. Treatment of burns in the first 24 hours: Simple and practical guide by answering 10 questions in a step-by-step form. World J Emerg Surg 2012;7:1–10 22583548 10.1186/1749-7922-7-13 PMC3506488

[25] Sawe HR, Mfinanga JA, Lidenge SJ, et al. Disease patterns and clinical outcomes of patients admitted in intensive care units of tertiary referral hospitals of Tanzania. BMC Int Health Hum Rights 2014;14:1–8 10.1186/1472-698X-14-26 PMC4204389 25245028

[26] Spiwak R, Lett R, Rwanyuma L, Logsetty S Creation of a standardized burn course for low income countries: Meeting local needs. Burns 2014;40:1292–1299 24685348 10.1016/j.burns.2014.01.007

[27] Klingberg A, Wallis LA, Hasselberg M, Yen P-Y, Fritzell SC Teleconsultation using mobile phones for diagnosis and acute care of burn injuries among emergency physicians: Mixed-methods study. JMIR mHealth uHealth 2018;6:e11076 30341047 10.2196/11076 PMC6231743

[28] Blom L, Laflamme L, Alvesson HM Expectations of medical specialists about image-based teleconsultation–A qualitative study on acute burns in South Africa. PLoS One 2018;13:e0194278 29543847 10.1371/journal.pone.0194278 PMC5854403

[29] Kifle M, Payton FC, Mbarika V, Meso P Transfer and adoption of advanced information technology solutions in resource-poor environments: The case of telemedicine systems adoption in ethiopia. Telemed J E Health 2010;16:327–343 20406120 10.1089/tmj.2009.0008

[30] Hill RJ, Fishbein M, Ajzen I Belief, attitude, intention and behavior: An introduction to theory and research. Contemp Sociol 1977;6:244

[31] Gagnon M-P, Desmartis M, Labrecque M, et al. Systematic review of factors influencing the adoption of information and communication technologies by healthcare professionals. J Med Syst 2012;36:241–277 20703721 10.1007/s10916-010-9473-4 PMC4011799

[32] Park Y, Chen J V Acceptance and adoption of the innovative use of smartphone. Ind Manag Data Syst 2007;107:1349–1365

[33] Goswami A, Dutta S Gender differences in technology usage—A literature review. Open J Bus Manag 2016;4:51–59

[34] Yarbrough AK, Smith TB Technology acceptance among physicians: A new take on TAM. Med Care Res Rev 2007;64:650–672 17717378 10.1177/1077558707305942

[35] Alexandre B, Reynaud E, Osiurak F, Navarro J Acceptance and acceptability criteria: A literature review. Cogn Technol Work 2018;20:165–177

[36] Nikolic A, Wickramasinghe N, Claydon-Platt D, Balakrishnan V, Smart P The use of communication apps by medical staff in the australian health care system: Survey study on prevalence and use. JMIR Med Inform 2018;6:e9 29426813 10.2196/medinform.9526 PMC5889814

[37] Hampshire K, Porter G, Mariwah S, et al. Who bears the cost of “informal mhealth”? Health-workers' mobile phone practices and associated political-moral economies of care in Ghana and Malawi. Health Policy Plan 2017;32:34–42 27476501 10.1093/heapol/czw095 PMC5886236

[38] Ayiasi RM, Atuyambe LM, Kiguli J, Orach CG, Kolsteren P, Criel B Use of mobile phone consultations during home visits by Community Health Workers for maternal and newborn care: Community experiences from Masindi and Kiryandongo districts, Uganda. BMC Public Health 2015;15:1–13 26084369 10.1186/s12889-015-1939-3 PMC4471930

[39] Hossain N, Yokota F, Sultana N, Ahmed A Factors influencing rural end-users' acceptance of eHealth in developing countries: A study on Portable Health Clinic in Bangladesh. Telemed J E Health 2018;25:221–229 29664328 10.1089/tmj.2018.0039 PMC6441281

[40] Venkatesh M, Davis D User acceptance of information technology: Toward a unified view. MIS Q 2003;27:425–478

